# Human nerve distribution and density around the carotid artery bifurcation

**DOI:** 10.1007/s00392-024-02419-0

**Published:** 2024-02-26

**Authors:** Helge Struthoff, Lucas Lauder, Jan M. Federspiel, Mathias Hohl, Michael Böhm, Thomas Tschernig, Felix Mahfoud

**Affiliations:** 1 Internal Medicine, Saarland University Medical Center, Kirrberger Straße, 66421 Homburg, Saar Germany; 2 Institute of Legal Medicine, Saarland University Medical Center, Kirrberger Straße, 66421 Homburg, Saar Germany; 3 Institute of Anatomy and Cell Biology, Saarland University Medical Center, Kirrberger Straße, 66421 Homburg, Saar Germany; 4 Institute for Medical Engineering and Science, Massachusetts Institute of Technology, Cambridge, MA USA

## Introduction

Modulation of the autonomic nervous system represents an attractive treatment target for cardiovascular diseases, including hypertension, heart failure and atrial fibrillation [1]. Stretch-sensitive baroreceptors in the carotid sinus contribute to short- and long-term blood pressure regulation. Increased wall strain due to high blood pressure increases the activity of arterial baroreceptors and thereby reduces sympathetic outflow and total peripheral resistance [3, 5]. Stimulation of the carotid baroreflex using electrical properties or a stent-like device is currently under clinical investigation in heart failure and hypertension [1]. Detailed knowledge about the nerves running along the carotid arteries is essential to identify attractive treatment targets and locations and to further refine existing technologies and techniques. Here, we analyzed the nerves traveling along the human carotid arteries.

## Materials and methods

**Fig. 1:**
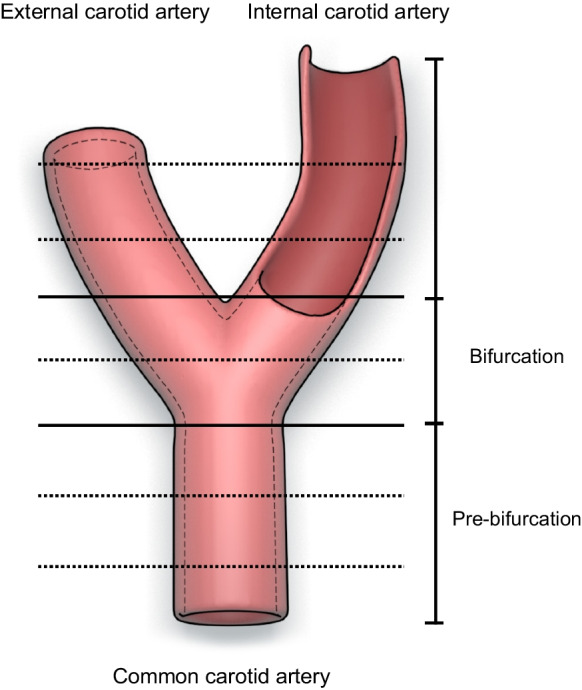
Schematic illustration of the carotid arteries. The black, horizontal lines indicate the localization of the three segments. The black, horizontal, dashed lines show the localization of the histological slides

A total of 17 human carotid arteries were excised from 9 body donors (2 females, 7 males, mean age 85 years) within 24 h post-mortem (Ethical Committee permission number 162/20). Tissue samples of different regions such as the common carotid artery (CCA pre-bifurcation, CCA bifurcation) and the internal (ICA) and external carotid artery (ECA) with adjacent tissue were excised (Fig. 1). Samples were fixed in buffered 4% formaldehyde and embedded in paraffine. Immunofluorescence and digital evaluation were performed as described elsewhere [4]. Briefly, paraffine sections and immunofluorescence stains (S-100 from Dako (IR504), for all nerves, tyrosine hydroxylase [TH] from Abcam (ab112) for efferent nerves, calcitonin gene-related peptide [CGRP] from Abcam (ab47027) for afferent nerves) were prepared from the specimens. A secondary anti-rabbit-FITC from Biozol (711–095-152) was used for the detection of the primary antibodies. We aimed to assess the density (nerves per cm^2^, from proximal to distal) and distribution of the nerves, the nerve size (measured at the smallest diameter; assigned to small (35–69 µm), middle (70–140 µm) and large (> 140 µm) and the distance of the nerves from the endothelium. To further investigate efferent and afferent characteristics, 430 nerves in 15 slides were measured using TH-staining, and 152 nerves in 14 slides were analyzed in TH- and CGRP-staining. Stained areas of identical nerves were evaluated on two consecutive sections (one labeled with TH and one with CGRP). Data were presented as TH/CGRP quotient.

The software SPSS Statistics version 27.0 (IBM) was used for statistical analysis. For examination of normal distribution, histograms and the Shapiro–Wilk test were used. All data were calculated as mean ± SD. Arteries, regions, and quadrants were compared using the Kruskal–Wallis test or the Mann–Whitney-U test. The Wilcoxon signed-rank test was used on the calculated mean values of carotid arteries to compare the left and right carotid arteries. For statistical significance, a 2-tailed p-value < 0.05 was provided.

## Results

**Fig. 2:**
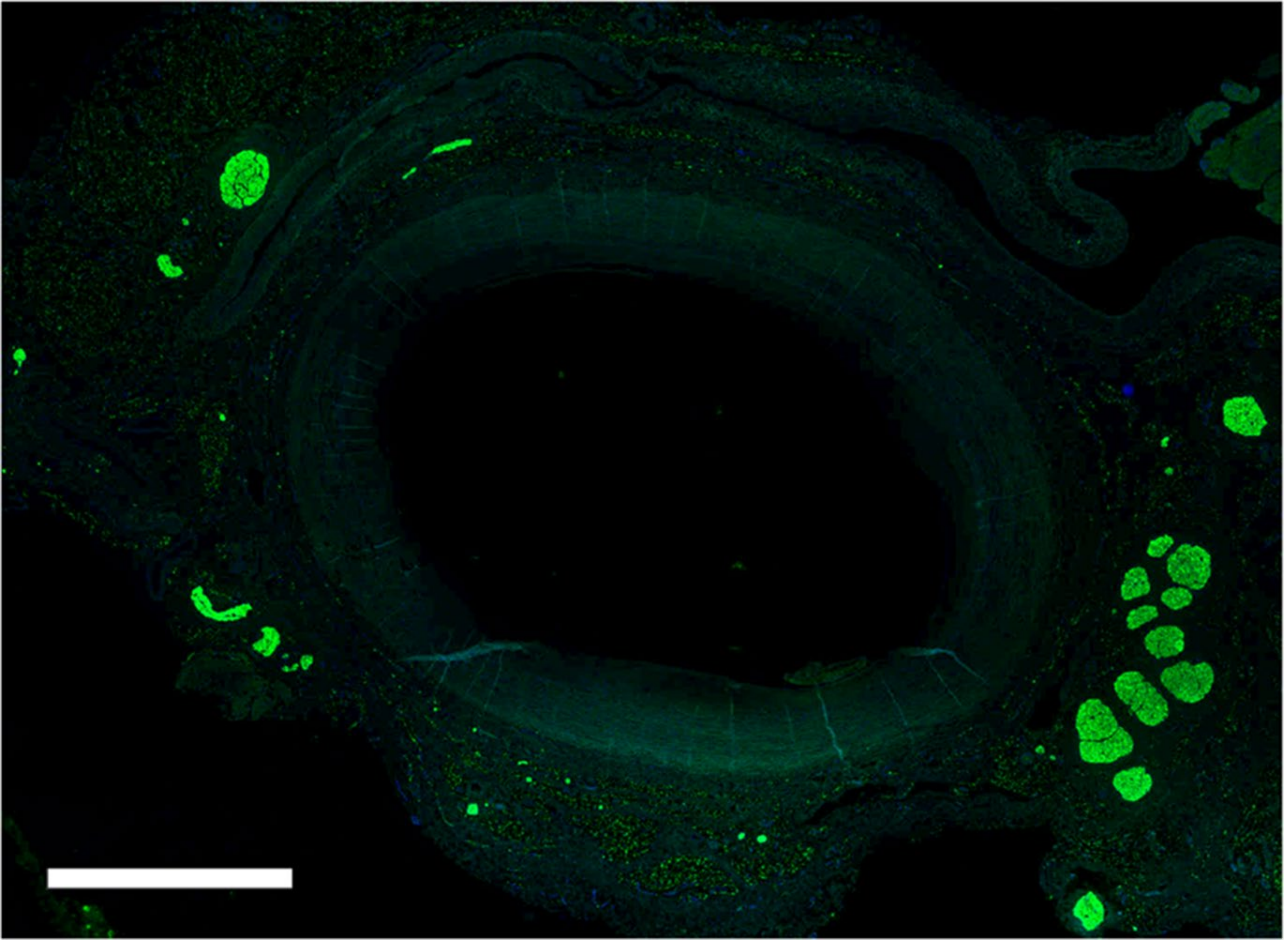
Section of the proximal common carotid artery (pre-bifurcation) stained with primary antibody anti S100 and secondary antibody anti-Rabbit FITC. The bar represents 3000 µm

The average diameter of the investigated vessels was: CCA pre-bifurcation 6.3 ± 1.1 mm, bifurcation 7.9 ± 2.3 mm, ICA 5.1 ± 1.3 mm, and ECA 3.6 ± 1.2 mm, respectively. In 15 of 17 arteries (88%), arteriosclerotic plaques were detected. A representative figure with staining against S100 was presented in Fig. 2. The overall nerve density was 46 ± 51 nerves/cm^2^. There was no statistically significant difference in nerve density when comparing the left and right carotid arteries (47 ± 54 vs. 46 ± 48 nerves/cm^2^; *p* = 1.0), as well as when comparing the ICA and ECA (48 ± 50 vs. 64 ± 63 nerves/cm^2^; *p* = 0.076). The number of nerves was lowest in the bifurcation region and highest in the ICA/ECA (Table 1). The density of nerve fibers per cm^2^ increased from the pre-bifurcation and bifurcation regions to the ICA/ECA (33 ± 38, 35 ± 33, 58 ± 60 nerves/cm^2^, *p* < 0.001 for CCA pre-bifurcation vs. ICA/ECA, *p* = 1.000 for CCA pre-bifurcation vs. CCA bifurcation and *p* = 0.127 for CCA bifurcation vs. ICA/ECA). The density of nerve fibers was not significantly different between the quadrants (range from 37 ± 37 to 55 ± 64 nerves/cm^2^).

**Table 1:** Number of nerves and distance between the inner side of the nerves and the endothelium

	Pre-bifurcation	Bifurcation	ICA/ECA	Total
Number of nerves	792	408	1714	2914
Lumen-to-nerve distance, mm	2.9 ± 1.4	2.9 ± 1.4	2.5 ± 1.5	2.6 ± 1.6

Data are frequencies or mean ± standard deviations (SD)

**Fig. 3:**
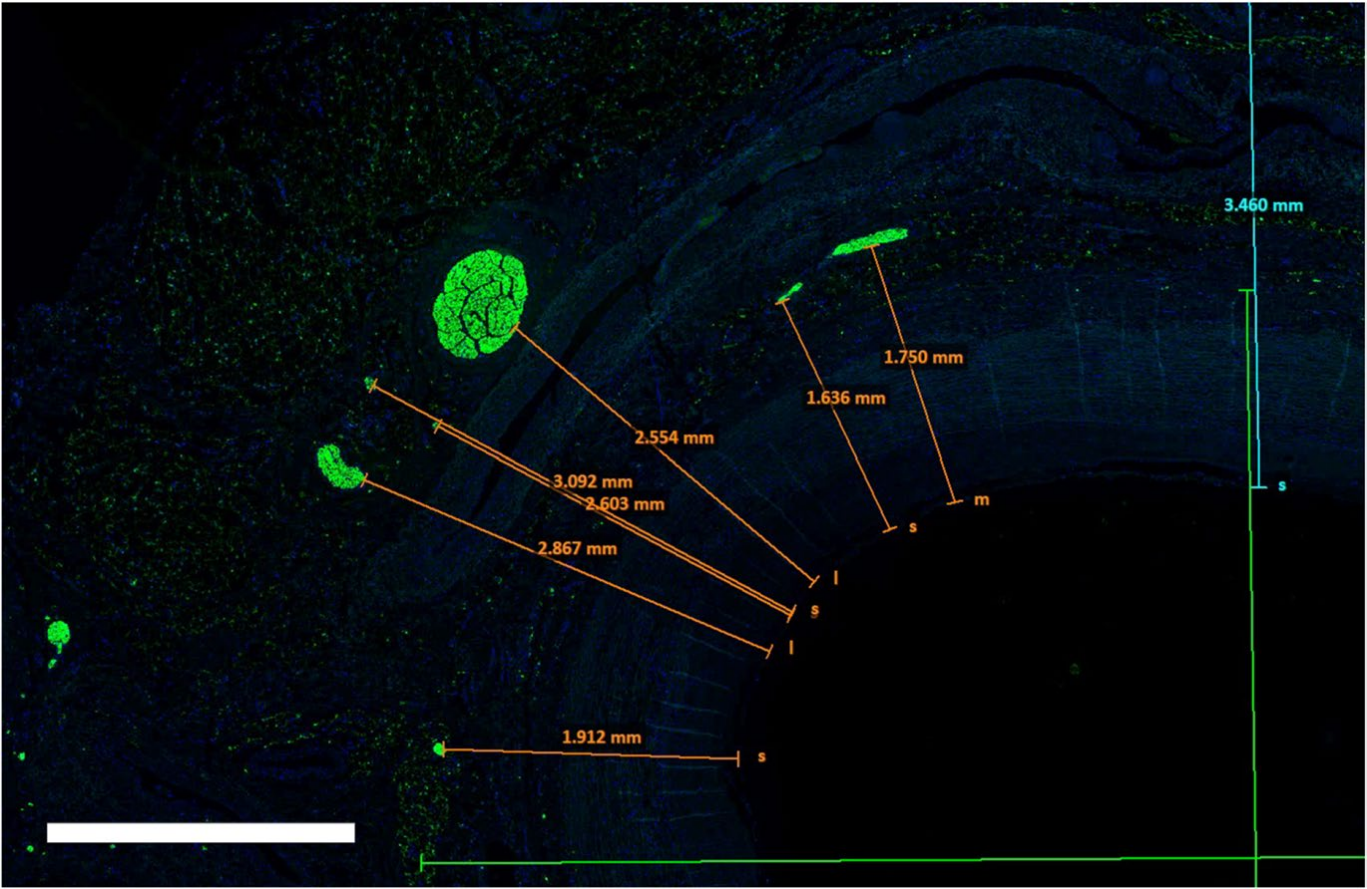
Screenshot from the evaluation procedure to visualize examples of the measurements of distances from the endothelium to the nerves. This example shows an S100-stained slide from the proximal common carotid artery (pre-bifurcation). The bar represents 2000 µm

**Fig. 4:**
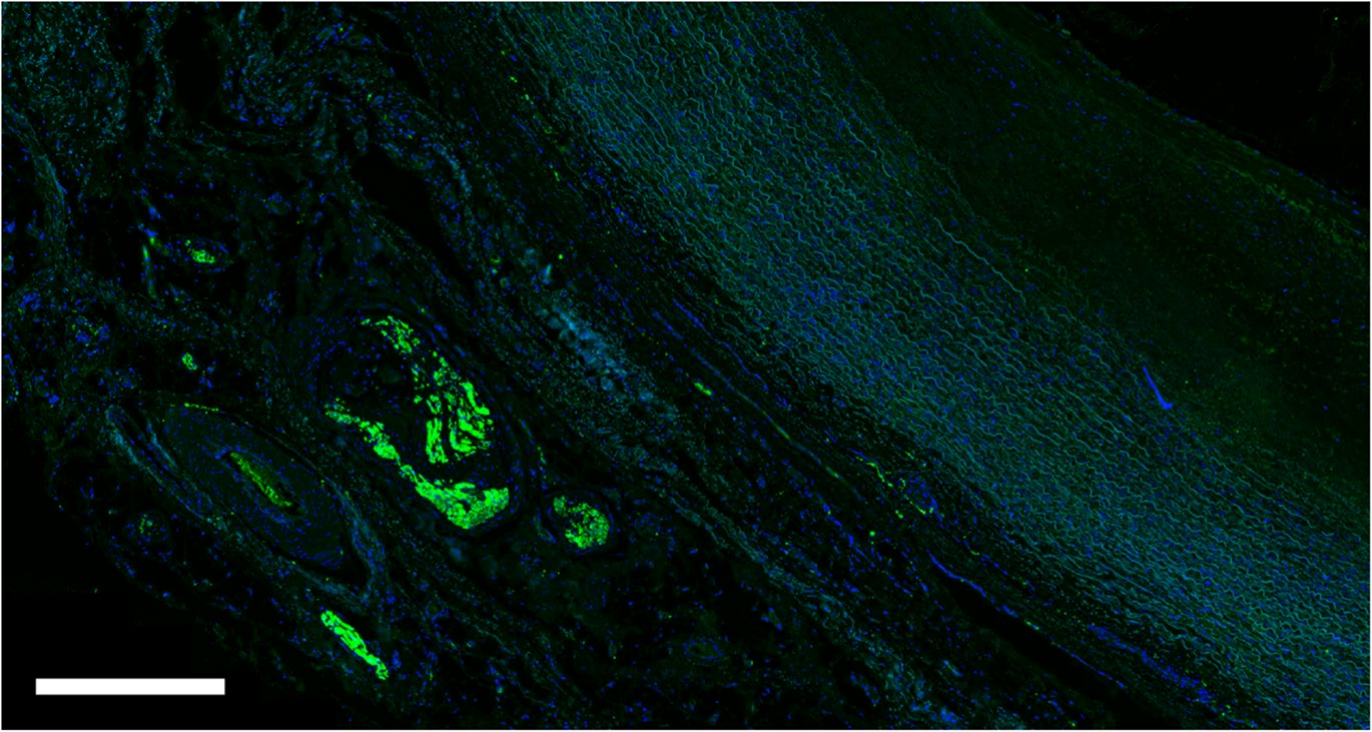
Section of the proximal common carotid artery (pre-bifurcation) stained with primary antibody anti TH and secondary antibody anti-Rabbit FITC. The bar represents 500 µm

**Fig. 5:**
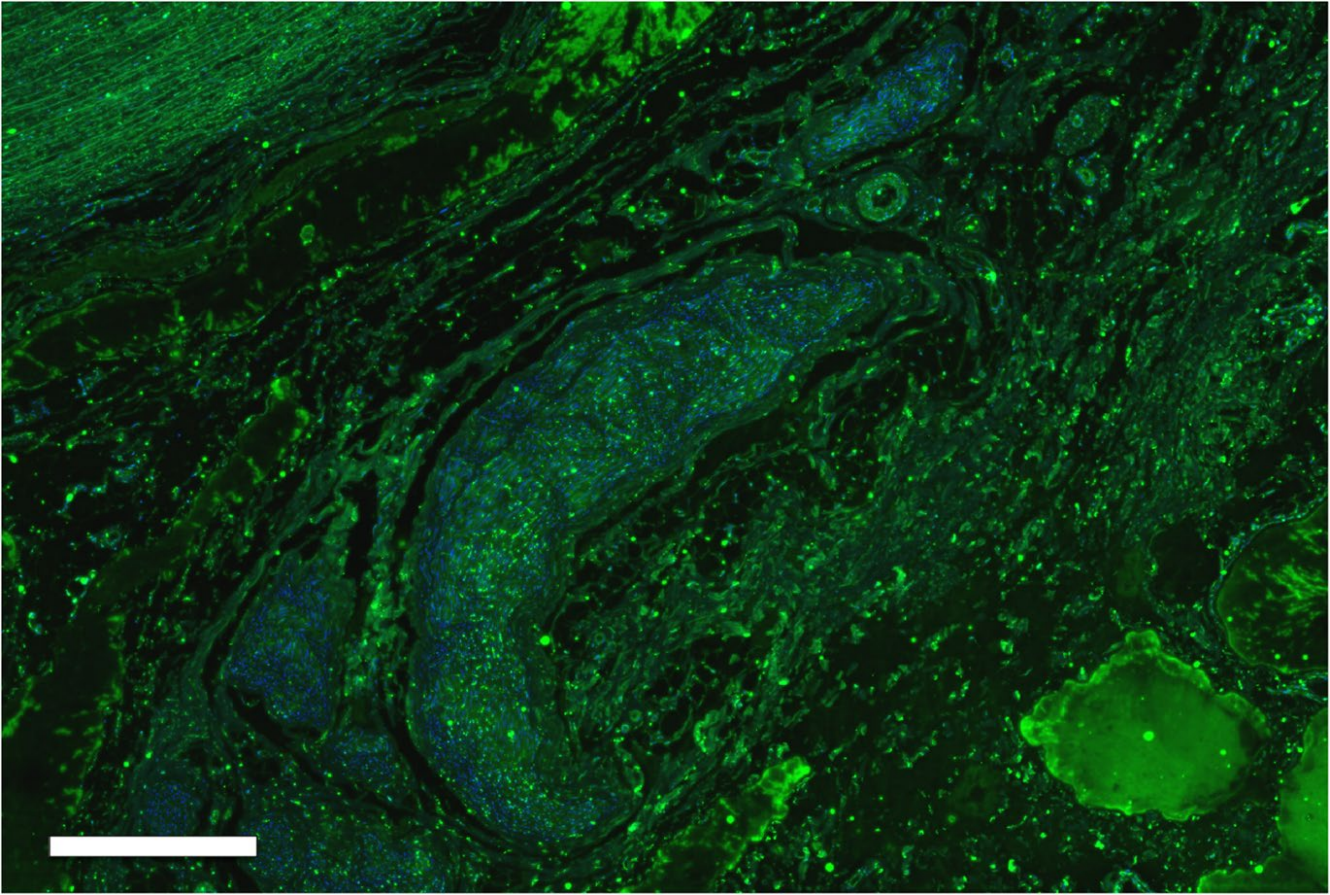
Section of the carotid artery (bifurcation) stained with primary antibody anti CGRP and secondary antibody anti-Rabbit FITC. The bar represents 500 µm

**Fig. 6:**
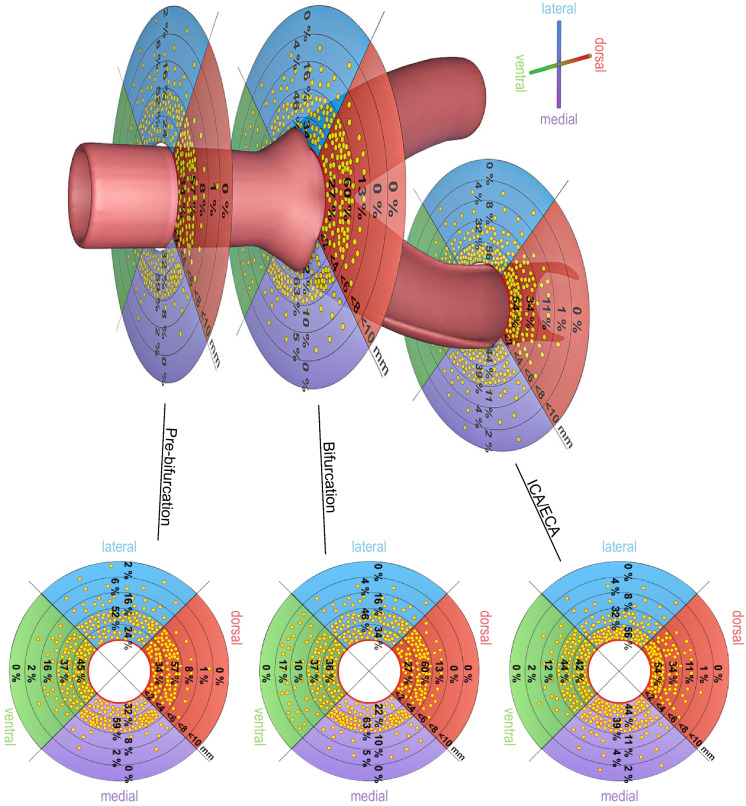
Schematic illustrations of the results. Based on mean values of S100-staining in the three sections. Every dot represents one percent of nerves including all sizes in the quadrants dorsal, lateral, ventral and medial

The proportion of large nerves was 39% in the CCA pre-bifurcation, 34% in CCA bifurcation and 22% in ICA/ECA. The proportion of small nerves increased from CCA pre-bifurcation (34%) to CCA bifurcation (38%) and ICA/ECA (53%). The mean distance from the lumen was equal in the CCA pre-bifurcation and CCA bifurcation region but markedly decreased in the ICA/ECA segments. An example of the evaluation of distances was presented in Fig. 3. We identified 430 nerves in 15 TH-stained slides (Fig. 4). These nerves showed a lumen-to-nerve distance of 2.6 ± 1.1 mm, 2.4 ± 0.7 mm, and 2.5 ± 1.1 mm in the pre-bifurcation, bifurcation, and ICA/ECA segments, respectively. In 14 representative slides, 152 TH-stained nerves were matched with the corresponding nerve in consecutive CGRP-stained slides (Fig. 5). The overall TH-positive area was 90%, and the CGRP-positive area was 10% (TH/CGRP-quotient was between 39 and 45). Small nerves (30%) had a higher TH/CGRP quotient (42 ± 39) than middle (31%) and large (39%) nerves (39 ± 50 and 32 ± 58, *p* < 0.001). In addition, 21 lymph nodes and 13 ganglia were found along the carotid arteries. A summary of main results was additionally presented in Fig. 6.

## Discussion

In this study, nerves converged to the carotid artery’s lumen from proximal (CCA pre-bifurcation) to distal (ICA/ECA), resulting in the lowest lumen-to-nerve distance in the ICA/ECA segments. Moreover, the number of nerves was highest in the ICA/ECA segments as well as in the medial and dorsal quadrants. A trend was observed to a higher density of nerves in the ECA as compared to ICA which might influence approaches of selective baroreceptor activation in the future. Efferent nerves occurred predominantly, and the ratio of efferent to afferent nerves also increased from proximal to distal. A limitation in the presented pilot study is the missing differentiation between nerve fibers, nerve bundles and complete nerves with different layers of sheaths. This would require an additional and very extended and complex study. The presences of atherosclerotic plaques might influence both, the nerve morphology as well as the efficacy of electrical baroreflex activation therapy. Plaques were found in all of the body donors and most of the investigated arteries (88%) but their influence on the morphology of nerves could not be evaluated systematically because of the overall limited number of samples. Nevertheless, we have not found significant alterations in the samples with atherosclerotic plaques regarding the number of nerves. In addition, the presence of hypertension might be of importance as a cause or as a consequence of morphological alterations. Because of the limited number of cases and the challenge of precisely defining hypertension in body donors this aspect was not included in our study.

Baroreceptor activation therapy (BAT) is still considered an investigational treatment option due to the lack of outcome data, but evidence has shown positive effects on exercise capacity and quality of life [2]. Nerves involved in the baroreceptor reflex have been studied before using immunohistochemistry [6, 8]. Toorop and colleagues found VGLUT2 and PGP 9.5 immunoreactivity in the adventitia of the carotid arteries. Nerve density was highest in the medial wall of the proximal first cm of the internal carotid artery. Furthermore, adventitial stripping of the internal carotid artery in patients with carotid sinus syndrome was performed to prevent syncope in those patients [7]. Interestingly, the blood pressure was not significantly affected. One could argue that Horner syndrome might occur in rare cases, but this complication was not reported.

Device-based approaches have been investigated for the treatment of cardiovascular diseases, including hypertension, heart failure, and atrial fibrillation. Several approaches target sympathetic nerves along the carotid arteries. These include BAT with a pacemaker-like device, endovascular baroreflex amplification therapy by the unilateral, endovascular implantation of a self-expanding nitinol stent in the proximal internal carotid artery, and transvenous carotid body ablation [1]. The autonomic nervous system is largely affected by peripheral chemoreceptors, of which the carotid body is the dominant peripheral chemoreceptor in humans, and the baroreceptor reflex. In response to increased wall strain, the firing rate of baroreceptors increases, resulting in reduced sympathetic outflow and total peripheral resistance. Of note, compared with other vessels, such as the renal arteries, the nerve density along the carotid arteries is about 30% lower (46 vs. 66 nerves per cm^2^) [4].

Taken together, as the nerves converged to the carotid artery’s lumen and the ratio of efferent to afferent nerves increased from proximal (pre-bifurcation) to distal (ICA/ECA), the ICA/ECA segments may represent an attractive treatment area if efferent nerve signaling is targeted.

## Data Availability

Data available on request from the authors.

## References

[1] Lauder L, Azizi M, Kirtane AJ, Bohm M, Mahfoud F (2020) Device-based therapies for arterial hypertension. Nat Rev Cardiol 17:614–628 32286512 10.1038/s41569-020-0364-1

[2] Malangu B, Lanier GM, Frishman WH (2021) Nonpharmacologic treatment for heart failure: a review of implantable carotid baroreceptor stimulators as a therapeutic option. Cardiol Rev 29:48–53 32282391 10.1097/CRD.0000000000000307

[3] McBryde FD, Abdala AP, Hendy EB, Pijacka W, Marvar P, Moraes DJ, Sobotka PA, Paton JF (2013) The carotid body as a putative therapeutic target for the treatment of neurogenic hypertension. Nat Commun 4:2395 24002774 10.1038/ncomms3395

[4] Struthoff H, Lauder L, Hohl M, Hermens A, Tzafriri AR, Edelman ER, Kunz M, Bohm M, Tschernig T, Mahfoud F (2023) Histological examination of renal nerve distribution, density, and function in humans. EuroIntervention 19(7):612–620 37501502 10.4244/EIJ-D-23-00264 PMC10493771

[5] Timmers HJ, Wieling W, Karemaker JM, Lenders JW (2003) Denervation of carotid baro- and chemoreceptors in humans. J Physiol 553:3–11 14528027 10.1113/jphysiol.2003.052415 PMC2343492

[6] Toorop RJ, Ousrout R, Scheltinga MR, Moll FL, Bleys RL (2013) Carotid baroreceptors are mainly localized in the medial portions of the proximal internal carotid artery. Ann Anat 195:248–252 23452666 10.1016/j.aanat.2012.12.001

[7] Toorop RJ, Scheltinga MR, Bender MH, Charbon JA, Huige MC, Moll FL, Bruijninckx CM (2009) Effective surgical treatment of the carotid sinus sindrome. J Cardiovasc Surg (Torino) 50:683–686 18948872

[8] Toorop RJ, Scheltinga MR, Moll FL, Bleys RL (2009) Anatomy of the carotid sinus nerve and surgical implications in carotid sinus syndrome. J Vasc Surg 50:177–182 19563966 10.1016/j.jvs.2009.03.029

